# Steroid insensitive respiratory inflammation - an acute tobacco smoke model

**DOI:** 10.1186/1476-9255-10-S1-P30

**Published:** 2013-08-14

**Authors:** Hemanth Ramaprakash, Jason Riley, Navin L Rao

**Affiliations:** 1 Janssen Research and Development, LLC. San Diego, CA 92121, USA

## 

Steroid insensitive inflammation is implicated with respiratory diseases such as Chronic Obstructive Pulmonary Disease (COPD), and asthma. One of the barriers to effective target screening in this area is the lack of in vivo models that are both relevant and of short duration. We present here our work developing an in-house model for steroid insensitive inflammation that involves the use of a major causative agent of neutrophilic inflammation and respiratory disease, tobacco smoke. We examined the effects of 4 days of cigarette smoke exposure in both Balb/c and C57BL/6 mice. Our investigations of bronchoalveolar lavage (BAL) fluid, lung tissue, and cellular analysis in our murine model indicate strain specific effects in response to acute tobacco smoke and that the neutrophil and macrophage associated cytokines such as KC/GRO, IL-1b, MCP-1, and IL-17A are significantly elevated in the model. Further, acute smoke induced inflammation while unresponsive to steroids such as dexamethasone, is also unresponsive to modulation by known neutrophilic chemotactic mechanisms such as LTB_4_, IL17A and KC. Examination of the cellular components of BAL and lung revealed neutrophilic infiltrate early that resolves with a dominant macrophage component during the resolution phase of inflammation. Further, genechip analysis of lungs from mice exposed to acute smoke demonstrated that 58 unique genes were significantly elevated in response to tobacco smoke and that many are macrophage associated genes observed in respiratory disease.

